# Liking of salt is associated with depression, anxiety, and stress

**DOI:** 10.1093/chemse/bjad038

**Published:** 2023-09-20

**Authors:** Celeste Ferraris, Christopher J Scarlett, Tamara Bucher, Emma L Beckett

**Affiliations:** School of Environmental and Life Sciences, The University of Newcastle, Ourimbah, NSW 2258, Australia; School of Environmental and Life Sciences, The University of Newcastle, Ourimbah, NSW 2258, Australia; Hunter Medical Research Institute, Newcastle, NSW 2305, Australia; School of Environmental and Life Sciences, The University of Newcastle, Ourimbah, NSW 2258, Australia; Priority Research Centre for Physical Activity and Nutrition, The University of Newcastle, Callaghan, NSW 2308, Australia; Hunter Medical Research Institute, Newcastle, NSW 2305, Australia; School of Environmental and Life Sciences, The University of Newcastle, Ourimbah, NSW 2258, Australia; Priority Research Centre for Physical Activity and Nutrition, The University of Newcastle, Callaghan, NSW 2308, Australia; Hunter Medical Research Institute, Newcastle, NSW 2305, Australia

**Keywords:** DASS-21, foods, mood, sodium, survey, taste

## Abstract

Early research has shown variations in salt taste qualities in depression, anxiety, and stress. These studies evaluated changes to salt taste intensity and liking (pleasantness) of salt solutions but not of salty foods. Therefore, an Australian population survey (*n* = 424) was conducted where participants rated recalled intensity and liking of salt index foods and completed the Depression, Anxiety, and Stress Scale (DASS-21) to measure these states. Standard least squares regression (post hoc Tukey’s HSD) compared means between groups, and nominal logistic regression assessed differences in distributions between categories. Higher salt liking was found in participants with DASS-21 scores indicative of severe depression (68.3 vs. 60.0, *P* = 0.005) and severe anxiety (68.4 vs. 60.0, *P* = 0.001) in comparison to those with normal scores, in all models. Higher salt liking was found in participants with DASS-21 scores indicative of moderate stress (67.7 vs. 60.2, *P* = 0.009) in the unadjusted model only. Higher salt liking was found in females with DASS-21 scores indicative of anxiety and stress, and in males with indicative depression and anxiety. No relationships between salt taste intensity ratings and the mood states were found. Results indicate that liking salty foods is positively correlated with depression, anxiety, and stress scores. Further research on the relationships between salt liking and intake of salt and salty foods, and the biological mechanisms of these mood states are needed to direct the application of findings toward potential new risk assessment measures, dietary interventions, or therapeutics.

## 1. Introduction

Depression and anxiety are increasingly common health conditions (Australian Bureau of Statistics 2018; National Mental Health Commission 2018) and leading contributors to the global burden of disease (James et al. 2018). Since the start of the COVID-19 pandemic, there has been an estimated 27.6% increase in depressive disorders and a 25.6% increase in anxiety disorders worldwide (Santomauro et al. 2021). Psychological stress or distress is a contributing factor to depression and anxiety (Hankin et al. 2004) and is fast becoming a significant health concern alone (Mariotti 2015; Devi et al. 2021). Diagnosis of depression, anxiety, or stress can be challenging due to unknown etiologies (Devane et al. 2005; Bystritsky et al. 2013) and their often-comorbid appearance (Groen et al. 2020; Kalin 2020). In addition, treatment effectiveness can be widely variable in depression (Khan et al. 2002; Fournier et al. 2010; Porcelli et al. 2011; Linde et al. 2015; Cipriani et al. 2018) and anxiety (Baldwin et al. 2011; Locher et al. 2017; Slee et al. 2019), and treatment side effects can be significant and lead to medication non-adherence (Gartlehner et al. 2008; Amick et al. 2015; Locher et al. 2017; Slee et al. 2019). Therefore, further elucidation of the complex etiologies and treatment methods are needed.

Diet is a well-known modifier of mental health outcomes. Diets low in whole foods are significantly related to increased risks of anxiety (McMartin et al. 2013; Nguyen et al. 2017; Brookie et al. 2018) and depression (McMartin et al. 2013; Liu et al. 2016; Brookie et al. 2018). These relationships are bi-directional and cyclical in that mental health conditions influence eating habits and dietary choices, which then impact mental health (Gardner et al. 2014; Farhangi et al. 2018). The interest in food and mood relationships has led to many studies evaluating interventions aimed at increasing diet quality to improve mental health (Opie et al. 2015; Ramsden et al. 2015; Firth et al. 2019). However, in practice, improving an individual’s diet quality is challenging and ongoing adherence is low (Kesse-Guyot et al. 2011; Kris‐Etherton et al. 2020). Research on taste qualities has provided knowledge on how we choose the foods we eat (Drewnowski et al. 1999; Beckett et al. 2014; Ervina et al. 2021; Pagliarini et al. 2021) and may be valuable in improving mental health outcomes from dietary interventions.

Variations in taste perception contribute to individual food preferences (Vink et al. 2020; Ervina et al. 2021) and therefore may modulate the intake of foods (Liem and Russell 2019) related to mental health (McMartin et al. 2013; Nguyen et al. 2017; Brookie et al. 2018). Beyond whole foods, the substrates within are also well-studied for their roles in mental health (Muscaritoli 2021). While the sensory experience of taste influences food choice (Vink et al. 2020; Ervina et al. 2021), there may also be interactions between taste and food substrates that have physiological functions or impacts on mental health. For example, diets associated with poor mental health are typically high in discretionary foods which are also high in sodium (Rees et al. 2021). Salt intake has recently been associated with depression and anxiety (Grippo et al. 2006; Morris et al. 2006, 2008, 2010; Goldstein and Leshem, 2014; Özdemir et al. 2014; Sawant et al. 2019; Bojdani et al. 2020), and correcting low serum sodium levels can improve symptoms of depression (Sawant et al. 2019; Bojdani et al. 2020). Variations in aspects of salt taste such as thresholds, intensity, and liking, can impact salt intake (Hayes et al. 2010; Pilic et al. 2020; Veček et al. 2020) and therefore, these taste qualities have been used as markers in mood research (Heath et al. 2006; Al’Absi et al. 2012; Ileri-Gurel et al. 2013; Büttner et al. 2015).

Two studies in otherwise healthy populations found people with high trait anxiety had higher salt taste thresholds (Heath et al. 2006; Ileri-Gurel et al. 2013). One of these studies induced stress in people with trait anxiety and found that salt taste thresholds increased further (Ileri-Gurel et al. 2013). Variations in salt taste intensity have also been studied in stress, where higher stress levels (Itoh et al. 2022) and a rise in the stress biomarker cortisol (Al’Absi et al. 2012) were associated with a reduction in the perceived intensity of salty solutions. As secondary outcomes in a study exploring biomarkers in depression treatment, lower salt taste intensity and higher salt liking (pleasantness) were associated with a longer duration of depression (Büttner et al. 2015). Despite various interdependent relationships between aspects of salt taste, such as an inverted U-shape relationship between salt liking and intensity (Endrizzi et al. 2021), a negative association between salt taste intensity and salt liking (Spetter et al. 2010; Yeung et al. 2016; Chamoun et al. 2019; Yeung 2019; Veček et al. 2020), and a positive association between salt liking and salt taste thresholds (Hayes et al. 2010; Chamoun et al. 2019; Puputti et al. 2019; Pilic et al. 2020), not all aspects of salt taste have been studied in depression, anxiety, or stress.

Biologically, there are common processes between salt taste and mental health that suggest possible mechanisms for the relationships. Neurotransmitters such as serotonin (5-HT) and norepinephrine (NE) are modifiable factors that can alter salt taste (Heath et al. 2006; Roper and Chaudhari 2017; Smith et al. 2021) and mood (Dean and Keshavan 2017; Kaur and Singh 2017). Therefore, there may be salt taste-associated changes to neurotransmitters, impacting mental health. High salt concentrations may modulate mood through stimulation of the trigeminal nerve (TN) (Simon et al. 2008; Bigiani 2020, 2021) which also has an ameliorating effect on symptoms of depression (Schrader et al. 2011; Cook et al. 2013; Paulino Trevizol and Cordeiro 2016; Generoso et al. 2019) and anxiety (Trevizol et al. 2015; Freire et al. 2020). Other studies have demonstrated increased neuronal activity in the ventral pallidum (VP), the center of hedonic liking in the brain (Smith et al. 2009; Root et al. 2015), when salt liking increases under sodium depletion (Tindell et al. 2006) and in depression (Knowland et al. 2017). These common biological factors between salt taste, depression, and anxiety provide further support for additional research to be undertaken on their relationships.

While there is early evidence of changes to salt taste thresholds in anxiety and stress (Heath et al. 2006; Ileri-Gurel et al. 2013), there has been limited exploration of the relationships between these mood states and salt taste intensity and liking. In addition, changes to salt taste in depression have not been explored as a primary aim of research. The existing studies used sodium chloride solutions to assess aspects of salt taste (Heath et al. 2006; Al’Absi et al. 2012; Ileri-Gurel et al. 2013). However, humans consume salt in or with foods, and the relationships between these mood states and ratings of salt taste intensity and liking of salty foods have not been studied. Therefore, this cross-sectional study surveyed participants to determine their indicative level of depression, anxiety, and stress on the Depression, Anxiety, and Stress Scale (DASS-21), and their recalled perceived intensity and liking of salt index foods for correlation analysis. The outcomes of this research may be valuable in developing new risk assessment measures, dietary interventions, or therapeutics. For example, screening for mood conditions through measuring alterations to taste, or stimulating specific tastes to affect mood.

## 2. Materials and methods

### 2.1 *Study design and recruitment*

This cross-sectional study utilized survey data collected from Australian residents that were over 18 years of age. Participants were recruited through the University of Newcastle and social media channels (Facebook, LinkedIn, and Twitter) with snowball recruitment employed to engage further respondents. Qualtrics^XM^ (SAP, USA) software was used to create and administer the survey. The survey with 3 blocks (taste, mood, and demographics) was designed to be mobile and desktop computer friendly. Responses were collected over ~15 weeks (10 May 2021 to 24 August 2021). Consent was required for participation and ethics approval was granted by the Human Research Ethics Committee of the University of Newcastle (Reference No. H2021-0109).

### 2.2 Recalled salt taste intensity and liking

The first block of survey questions gathered data on recalled perceptions of salt taste intensity and liking for ten index foods: table salt, potato chips (plain), corn chips, bacon, french fries, processed meats, popcorn, hot dogs, nuts (salted), and crackers. These foods were identified as salt index foods in a taste intensity and liking recall study by Cornelis et al. 2017 that corroborated results across different study samples. Participants rated how intense they recalled the saltiness (salt intensity) of the index foods using a labeled magnitude scale (Green et al. 1996; Bartoshuk et al. 2004) and their liking (salt liking) of each food using a labeled affective magnitude scale (Schutz and Cardello 2001). For intensity, ratings of between 0 (barely detectable) and 100 (strongest imaginable) were possible, with 50 as moderate. For liking, ratings of between 0 (most disliked imaginable) and 100 (most liked imaginable) were possible, with 50 as neutral. Singular arbitrary unit ratings (0, 1, 2, 3 to 100) could be chosen with a left or right sliding bar on both the intensity and liking scales. If participants were unfamiliar with a food or did not eat it, they were able to select “not applicable.” The order of appearance of each index food was randomised between respondents. The ratings of salt intensity and liking for each salt index food were summed and the average was calculated to determine a mean score per participant.

### 2.3 Depression, anxiety, and stress

Depression, anxiety, and stress levels were estimated through completion of the DASS-21, a self-report screening tool for the severity of negative emotions experienced over the previous week (Lovibond and Lovibond 1995b). Reliability, validity, and internal consistency of the DASS-21 have been demonstrated in clinical (Clara et al. 2001; Gloster et al. 2008) and non-clinical adult samples (Henry and Crawford 2005; Norton 2007; Pezirkianidis et al. 2018). In addition, strong concurrent validity against other previously validated measures of depression, anxiety, and stress has been shown (Lovibond and Lovibond 1995a; Henry and Crawford 2005). The 3-factor structure applied in this study that is DASS-D for depression, DASS-A for anxiety, DASS-S for stress, has been validated in adult populations (Norton 2007; Gloster et al. 2008; Pezirkianidis et al. 2018), and across diverse racial and cultural groups (Norton 2007; Pezirkianidis et al. 2018).

Using 21 items, 7 items per mood state, participants rated the severity and frequency of their experienced depression, anxiety, and stress on a 4-point scale from 0 (did not apply to me at all) to 3 (applied to me very much or most of the time) (Lovibond and Lovibond 1995b). Following DASS guidelines, scores for each subscale were summed and multiplied by 2 to determine the final score, with subsequent placement of participants in the categories of normal, mild, moderate, severe, and extremely severe for each mood (Lovibond and Lovibond 1995b).

### 2.4 Demographics and taste acuity

The third block of questions collected demographic data including age, sex, height, weight, education, income, and smoking status. From height and weight data, body mass index (BMI) measures were calculated (Marfell-Jones 2001). Data on reported taste acuity was collected through participant selection from taste sense categories of normal, distorted, hypersensitive, partial loss, complete loss, or other, which allowed for the participant to specify. This enabled exclusion to be made for altered taste function that may be a result of medications (Doty et al. 2008), comorbidities, or other pathology (Bromley 2000).

### 2.5 Statistical analysis

The analyses of data were undertaken with JMP (Pro V.14.2.0; SAS Institute Inc., Cary, NC, USA 27513). Age and BMI were collected as continuous variables and then converted to categories for analysis. Age quartiles were created (18–30 years, 31–41 years, 42–52 years, and > 52 years), and BMI scores were categorized according to the World Health Organisation classifications (World Health Organisation 2021). The cohort characteristics and DASS-21 scores were described with categorical variable distributions (number, percentage). Mean scores for recalled salt intensity and liking across the 11 salt index foods were calculated as continuous data and distributions reported accordingly (mean, standard deviation (SD), min/max). The reliability and internal consistency of the DASS-21 and recalled salt taste intensity and liking scales were assessed with Cronbach’s alpha (Henry and Crawford 2005) using the acceptable threshold score of > 0.7 (Bland and Altman 1997; Tavakol and Dennick 2011).

For continuous outcome variables, least squares means were compared between groups using standard least squares regression analyses with Tukey’s post hoc tests. For categorical outcome variables, nominal logistic regression analyses were used to assess the differences in distributions between categories. *P* trends were reported to 1 significant figure and when intergroup *P*-values were statistically significant (< 0.05) they were noted by marking which means were significantly different. Adjustments were made for the potential confounders of age, sex, education, income, smoking status, and BMI, and the analyses were stratified by sex.

## 3. Results

### 3.1 Participant characteristics

From 715 participants who gave consent and commenced the survey, data were excluded where responses were incomplete (*n* = 202), respondents lived outside of Australia (*n* = 14), and if surveys were completed faster than half the median completion time of 509 s (*n* = 2). Participants reporting altered taste acuity were also excluded from the analyses (*n* = 64). To allow for robust statistical analyses, 9 participants who identified as “non-binary” or chose “prefer not to say” were excluded because of low numbers. The total sample size was 424.

The average age for the total sample was 41.3 (±SD 14.3) years, for females 40.7 (±SD 13.3) years, and for males 43.4 (±SD 17.9) years. The sample was predominantly female (80.2%) (Table 1). The mean BMI for the total sample was 27.4 kg/m^2^, for females 27.5 kg/m^2,^ and for males 27.0 kg/m^2^ placing all groups in the overweight category. Several categories within each variable were collapsed prior to statistical analyses due to low numbers. For household income, “< $20,000,” “$20,000 to $49,999” and “$50,000 to $79,999” were collapsed to “< $80,000”; and “$80.000 to $109,999” and $110,000 to $149,999’ were collapsed to “$80,000 to $149,999.” The largest proportion of participants had a household income of > $AUD 150,000 per year (29.3%) and this did not differ by sex (Table 1). For education, “< Year 12 or equivalent,” “Year 12 or equivalent” and “Technical and Further Education (TAFE) or technical diploma” (vocational training) were collapsed to “≤ TAFE or diploma.” The largest proportion of participants in the total sample and in females were educated at Bachelor’s degree level (35.6% and 38.2% respectively), and in males at Postgraduate level (31.0%) (Table 1). For smoking, “Currently smoking,” “Quit smoking < 12 months ago” and “Quit smoking > 12 months ago” were collapsed to “History of smoking.” Most participants in the total sample had never smoked (66.5%) and this did not differ by sex (Table 1).

**Table 1.** Key demographic variable distributions in the total sample, females and males.

Variable	Total	Females	Males
	*n* (%)	*n* (%)	*n* (%)
Age (years)			
18–30	109 (25.7)	85 (25.0)	24 (28.6)
31–41	106 (25.0)	92 (27.1)	14 (16.7)
42–52	115 (27.1)	93 (27.4)	22 (26.2)
> 52	94 (22.2)	70 (20.6)	24 (28.6)
Sex			
Females	340 (80.2)		
Males	84 (19.4)		
BMI (kg/m^2^)			
< 18.5 Underweight	5 (1.2)	4 (1.1)	1 (1.2)
18.5–24.9 Normal	173 (40.8)	144 (42.4)	29 (34.5)
25–29.9 Overweight	132 (31.1)	94 (27.7)	38 (45.2)
≥ 30 Obese	114 (26.9)	98 (28.8)	16 (19.1)
Household income (AUD/year)			
< $20,000	16 (3.8)	15 (4.4)	1 (1.2)
$20,000 to $49,999	42 (9.09)	37 (10.9)	5 (6.0)
$50,000 to $79,999	43 (10.1)	36 (10.6)	7 (8.3)
$80,000 to $109,999	71 (16.8)	54 (15.9)	17 (20.2)
$110,000 to $149,999	69 (16.3)	57 (16.8)	12 (14.3)
> $150,000	124 (29.3)	97 (28.5)	27 (32.1)
Prefer not to say	59 (13.9)	44 (12.9)	15 (17.9)
Education			
< Year 12 or equivalent	12 (2.8)	11 (3.2)	1 (1.2)
Year 12 or equivalent	62 (14.6)	42 (12.4)	20 (23.8)
TAFE or technical diploma	63 (14.9)	47 (13.8)	16 (19.1)
Bachelor’s degree	151 (35.6)	130 (38.2)	21 (25.0)
Postgraduate degree	134 (31.6)	108 (31.8)	26 (31.0)
Prefer not to say	2 (0.1)	2 (5.9)	0 (0)
Smoking			
Currently smoke	23 (5.4)	15 (4.4)	8 (9.5)
Quit smoking < 12 months ago	9 (2.1)	8 (2.4)	1 (1.2)
Quit smoking > 12 months ago	105 (24.8)	86 (25.3)	19 (22.6)
Never smoked	282 (66.5)	227 (66.8)	55 (65.5)
Prefer not to say	5 (1.2)	4 (1.2)	1 (1.2)

AUD, Australian dollars; TAFE, Technical and Further Education (vocational training). Percentages may not sum to 100% due to rounding.

### 3.2 Recalled salt intensity and liking distributions

The mean recalled salt intensity index score in the total sample was 54.3 (±SD 17.3), in females was 54.7 (±SD17.6), and in males was 52.6 (±SD 16.2) (Table 2). The mean recalled salt liking index score in the total sample was 61.5 (±SD 16.6), in females 61.1 (±SD 16.8), and in males 63.2 (±SD 15.9) (Table 2). There were no statistically significant differences between females and males in mean salt intensity (*P* = 0.3) or salt liking scores (*P* = 0.05). Both measures were normally distributed. Cronbach’s alpha values for the taste scales were 0.90 and 0.82, respectively, demonstrating high internal consistency.

**Table 2.** Mean recalled salt intensity and liking index scores in the total sample, females and males.

	Total	Females	Males
	Mean ±SD (min–max)	Mean ±SD (min–max)	Mean ±SD (min–max)
Salt intensity	54.3 ±17.3 (5.2–95.5)	54.7 ±17.6 (5.2–96.5)	52.6 ±16.2 (13.4–85.3)
Salt liking	61.5 ±16.6 (6.3–100.0)	61.1 ±16.8 (13.4–100.0)	63.2 ±15.9 (6.3–98.0)

SD, standard deviation.

### 3.3 DASS-21 distributions

Reliability and internal consistency values were above the acceptable Cronbach’s alpha threshold with DASS-D α at 0.93, DASS-A α at 0.82, and DASS-S α at 0.93. DASS-21 scores indicative of depression, anxiety, and stress were found in 37.3%, 34.6%, and 35.5% of the cohort respectively (Table 3). There was a statistically significant difference between females and males for DASS-S scores (χ^2^ = 11.9, *P* = 0.02) but not for DASS-D (χ^2^ = 9.4, *P* = 0.05) or DASS-A (χ^2^ = 4.3, *P* = 0.07) scores.

**Table 3.** DASS-21 distributions in the total sample, females and males.

DASS-21 category	Total	Females	Males
	*n* (%)	*n* (%)	*n* (%)
DASS-D			
Normal	266 (62.7)	217 (63.8)	49 (58.3)
Mild	58 (13.7)	49 (14.4)	9 (10.7)
Moderate	48 (11.3)	39 (11.5)	9 (10.7)
Severe	24 (5.7)	13 (3.8)	11 (13.1)
Extremely severe	28 (6.6)	22 (6.5)	6 (7.1)
DASS-A			
Normal	277 (65.3)	215 (63.2)	62 (73.8)
Mild	28 (6.6)	27 (7.9)	1 (1.2)
Moderate	62 (14.6)	50 (14.7)	12 (14.3)
Severe	26 (6.1)	21 (6.2)	5 (6.0)
Extremely severe	31 (7.3)	27 (7.9)	4 (4.8)
DASS-S			
Normal	273 (64.4)	213 (62.7)	60 (71.4)
Mild	54 (12.7)	44 (12.9)	10 (11.9)
Moderate	59 (13.9)	46 (13.5)	13 (15.5)
Severe	29 (6.8)	28 (8.2)	1 (1.2)
Extremely severe	9 (2.1)	9 (2.7)	0 (0)

DASS-D, depression; DASS-A, anxiety; DASS-S, stress. Percentages may not sum to 100% due to rounding.

Prior to further statistical analysis, several classifications within each DASS-21 mood grouping were collapsed due to low numbers. For DASS-D, DASS-A, and DASS-S “severe” and “extremely severe” were collapsed into “severe.” For DASS-A, “mild” and “moderate” were collapsed into “mild/moderate.”

### 3.4 Relationships between recalled salt taste liking, intensity, and potential confounding variables

When age was treated as a continuous variable, recalled salt liking decreased as age increased (*P* trend = 0.01; Tables 4 and 5). However, there were no statistically significant differences between the age groups (Tables 4 and 5). Salt liking increased as BMI increased (*P* trend = 0.02) but the intergroup means did not differ significantly (Tables 4 and 5). Salt liking was not associated with sex, income, education, or history of smoking (Tables 4 and 5).

**Table 4.** Relationships between recalled salt liking, salt intensity and confounding variables in the total sample.

Variable	Salt liking		Salt intensity	
	LSM ±SEM	*P* trend	LSM ±SEM	*P* trend
Age (years)				
18-30	64.4 ^a^ ±1.6	0.01	49.5 ^b^ ±1.6	0.008
31-41	63.9 ^a^ ±1.6		56.5 ^a^ ±1.7	
42-52	59.1 ^a^ ±1.5		55.3 ^ab^ ±1.6	
>52	58.6 ^a^ ±1.7		56.3 ^a^ ±1.8	
Sex				
Females	61.1 ^a^ ±0.9	0.6	54.7 ^a^ ±0.9	0.6
Males	63.2 ^a^ ±1.8		52.6 ^a^ ±1.9	
BMI (kg/m^2^)				
< 18.5	49.3 ^a^ ±7.4	0.02	41.0 ^a^ ±7.7	0.2
18.5-24.9	59.1 ^a^ ±1.3		54.5 ^a^ ±1.3	
25-29.9	63.7 ^a^ ±1.4		55.9 ^a^ ±1.5	
≥ 30	63.3 ^a^ ±1.5		52.8 ^a^ ± 1.6	
Income (AUD/year)				
< $80,000	61.8 ^a^ ±1.4	0.9	54.6 ^a^ ±1.4	0.03
$80,000–149,999	60.8 ^a^ ±1.6		57.1 ^ab^ ±1.7	
> $150,000	61.4 ^a^ ±1.5		54.5 ^ab^ ±1.6	
PNS	62.5 ^a^ ±2.1		48.8 ^b^ ±2.2	
Education				
≤ TAFE/Dip.	62.4 ^a^ ±1.4	0.3	51.9 ^b^ ±1.5	0.03
Bach.D	60.7 ^a^ ±1.3		53.6 ^ab^ ±1.4	
Postgrad.D	61.2 ^a^ ±1.4		58.0 ^a^ ±1.5	
PNS	77.9 ^a^ ±9.6		41.9 ^ab^ ±9.9	
Smoking				
History	62.4 ^a^ ±1.4	0.5	54.6 ^a^ ±1.5	0.8
Never	61.0 ^a^ ±1.0		54.3 ^a^ ±1.0	
PNS	67.0 ^a^ ±6.8		49.3 ^a^ ±7.1	

LSM, least squares mean; SEM, standard error of the mean; BMI, Body Mass Index; PNS, prefer not to say; TAFE, Technical and Further Education (vocational training); Dip., diploma; Bach.D., Bachelor’s degree; Postgrad.D., Postgraduate degree.

Values in the same row (within an analysis) denoted with the same letter are not significantly different from each other (Tukey’s HSD *p* < 0.05)

**Table 5.** Relationships between recalled salt liking, salt intensity, DASS-21 scores, and confounding variables in the total sample (normal taste acuity)

Variable	DASS-D (depression)					DASS-A (anxiety)				DASS-S (stress)				
	Normal	Mild	Mod	Severe	χ2 (*P*)	Normal	Mild/Mod	Severe	χ2 (*P*)	Normal	Mild	Mod	Severe	χ2 (*P*)
	*n* (%)					n (%)				n (%)				
Age (years)														
18-30	55 (13.0)	19 (4.5)	18 (4.3)	17 (4.0)	15.1 (0.09)	45 (10.6)	35 (8.3)	29 (6.8)	52.1 (<0.0001)	49 (11.6)	20 (4.7)	27 (6.4)	13 (3.1)	45.8 (<0.0001)
31-41	65 (15.3)	16 (3.8)	13 (3.1)	12 (2.8)		66 (15.6)	27 (6.4)	13 (3.1)		60 (14.1)	16 (3.8)	15 (3.5)	15 (3.5)	
42-52	76 (17.9)	14 (3.3)	12 (2.8)	13 (3.1)		86 (20.3)	19 (4.5)	10 (2.4)		85 (20.1)	13 (3.1)	11 (2.6)	6 (1.4)	
>52	70 (16.5)	9 (2.1)	5 (1.2)	10 (2.4)		80 (18.9)	9 (2.1)	5 (1.2)		79 (18.6)	5 (1.2)	6 (1.4)	4 (0.9)	
Sex														
Females	217 (51.2)	49 (11.6)	39 (9.2)	35 (8.3)	5.9 (0.1)	215 (50.7)	77 (18.2)	48 (11.3)	3.5 (0.2)	213 (50.2)	44 (10.4)	46 (10.9)	37 (8.7)	11.4 (0.01)
Males	49 (11.6)	9 (2.1)	9 (2.1)	17 (4.0)		62 (14.6)	13 (3.1)	9 (2.1)		60 (14.2)	10 (2.4)	13 (3.1)	1 (0.2)	
BMI (kg/m^2^)														
< 18.5	3 (0.7)	1 (0.2)	1 (0.2)	0 (0.0)	15.6 (0.08)	3 (0.7)	1 (0.2)	1 (0.2)	12.7 (0.05)	3 (0.7)	2 (0.5)	0 (0.0)	0 (0.0)	11.5 (0.2)
18.5-24.9	109 (25.7)	25 (5.9)	18 (4.3)	21 (5.0)		111 (26.2)	39 (9.2)	23 (5.4)		116 (27.4)	18 (4.3)	21 (5.0)	18 (4.3)	
25-29.9	95 (22.4)	15 (3.5)	8 (1.9)	14 (3.3)		100 (23.6)	21 (5.0)	11 (2.6)		89 (21.0)	19 (4.5)	16 (3.8)	8 (1.9)	
≥ 30	59 (13.9)	17 (4.0)	21 (5.0)	17 (4.0)		63 (14.9)	29 (6.8)	22 (5.2)		65 (15.3)	15 (3.5)	22 (5.2)	12 (2.8)	
Income (AUD/year)														
< $80,000	54 (12.7)	13 (3.1)	13 (3.1)	21 (5.0)	14.8 (0.1)	51 (12.0)	24 (5.7)	26 (6.1)	20.6 (0.002)	54 (12.7)	14 (3.3)	18 (4.3)	15 (3.5)	15.1 (0.09)
$80,000 to $149,999	94 (22.2)	16 (3.8)	16 (3.8)	14 (3.3)		95 (22.4)	32 (7.6)	13 (3.1)		95 (22.4)	20 (4.7)	12 (2.8)	13 (3.1)	
> $150,000	86 (20.3)	18 (4.3)	11 (2.6)	9 (2.1)		92 (21.7)	22 (5.2)	10 (2.4)		83 (19.6)	14 (3.3)	21 (5.0)	6 (1.4)	
PNS	32 (7.6)	11 (2.6)	8 (1.9)	8 (1.9)		39 (9.2)	12 (2.8)	8 (1.9)		41 (9.7)	6 (1.4)	8 (1.9)	4 (0.9)	
Education														
≤ TAFE/Dip.	70 (16.5)	20 (4.7)	17 (4.0)	30 (7.1)	23.8 (0.005)	75 (17.8)	33 (7.8)	29 (6.8)	20.3 (0.002)	78 (18.4)	19 (4.5)	27 (6.4)	13 (3.1)	11.7 (0.2)
Bach.D	96 (22.7)	22 (5.2)	20 (4.7)	13 (3.1)		97 (22.9)	38 (9.0)	16 (3.8)		96 (22.9)	19 (4.5)	21 (5.0)	15 (3.5)	
Postgrad.D	98 (23.1)	16 (3.8)	11 (2.6)	9 (2.1)		103 (24.3)	19 (4.5)	12 (2.8)		97 (22.9)	16 (3.8)	11 (2.6)	10 (2.4)	
PNS	2 (0.5)	0 (0)	0 (0)	0 (0)		2 (0.5)	0 (0.0)	0 (0.0)		2 (0.5)	0 (0.0)	0 (0.0)	0 (0.0)	
Smoking														
History	91 (21.5)	19 (4.5)	8 (1.9)	19 (4.5)	10.2 (0.1)	91 (21.5)	26 (6.1)	20 (4.7)	0.9 (0.9)	88 (20.8)	16 (3.8)	20 (4.7)	13 (3.1)	2.4 (0.9)
Never	171 (40.3)	39 (9.2)	40 (9.4)	32 (7.6)		183 (43.2)	63 (14.9)	36 (8.5)		182 (42.9)	37 (8.7)	39 (9.2)	24 (5.7)	
PNS	4 (0.9)	0 (0)	0 (0)	1 (0.2)		3 (0.7)	1 (0.2)	1 (0.2)		3 (0.7)	1 (0.2)	0 (0.0)	1 (0.2)	

Mod, moderate; BMI, Body Mass Index; AUD, Australian dollars; PNS, prefer not to say; TAFE, Technical and Further Education (vocational training); Dip., diploma; Bach.D, Bachelor degree; Postgrad.D, Postgraduate degree.

Values in the same row (within an analysis) denoted with the same letter are not significantly different from each other (Tukey’s HSD *p* < 0.05).

Recalled salt intensity varied with age (*P* trend = 0.008; (Tables 4 and 5). Salt intensity increased between the youngest age group (18–30 years) and those aged 31–41 years (14.1%, *P* = 0.02), and > 52 years (13.7%, *P* = 0.03) (Tables 4 and 5). Salt intensity was associated with income (*P* trend = 0.03) however the intergroup means did not differ significantly between those that reported income levels (Tables 4 and 5). Salt intensity was associated with education (*P* trend = 0.03; Tables 4 and 5). Those educated at postgraduate level recalled the saltiness of the index foods to be 11.8% more intense than those educated at TAFE level or below (*P* = 0.02; Tables 4 and 5). Salt intensity did not vary with sex, BMI, or history of smoking (Tables 4 and 5).

DASS-D scores indicative of depression were more likely in participants with lower education levels (≤ TAFE or technical diploma, *P* = 0.005; Tables 4 and 5). DASS-A scores indicative of anxiety were more likely in younger participants (18–30 years; *P* ≤ 0.0001), those with lower incomes (< $AUD 80,000/year, *P* = 0.002), and in those with lower education levels (≤ TAFE or technical diploma, *P* = 0.002) (Tables 4 and 5). DASS-S scores indicative of stress were more likely in younger participants (18–30 years, *P* ≤ 0.0001) and in females (*P* = 0.01) (Tables 4 and 5).

### 3.5 Relationships between DASS-21 and recalled salt taste liking and intensity in the total sample

Those with DASS-21 scores indicative of depression, anxiety, and stress had higher mean salt liking scores (Table 6), however, there were no associations between salt intensity and any of the mood states (Table 7).

**Table 6.** Recalled salt liking varies by DASS-21 scores indicative of depression, anxiety, and stress in the total sample (unadjusted and adjusted models).

DASS-21	Recalled salt liking							
	Unadjusted		Model 1		Model 2		Model 3	
	LSM ±SEM	*P* trend	LSM ±SEM	*P* trend	LSM ±SEM	*P* trend	LSM ±SEM	*P* trend
DASS-D								
** **Normal	60.0^b^ ±1.0	0.01	60.8^b^ ±1.2	0.03	66.0^b^ ±3.9	0.02	63.2^b^ ±4.3	0.04
** **Mild	61.5^ab^ ±2.2		61.6^ab^ ±2.3		67.1^ab^ ±4.5		64.3^ab^ ±4.8	
** **Moderate	62.7^ab^ ±2.4		62.5^ab^ ±2.5		68.6^ab^ ±4.6		65.4^ab^ ±4.9	
** **Severe	68.3^a^ ±2.3		68.3^a^ ±2.3		74.0^a^ ±4.5		70.7^a^ ±4.8	
DASS-A								
** **Normal	60.0^b^ ±1.0	0.002	61.1^b^ ±1.1	0.01	66.4^b^ ±3.9	0.01	63.4^b^ ±4.3	0.03
** **Mild/Moderate	62.1^ab^ ±1.8		62.3^ab^ ±1.9		67.8^ab^ ±4.3		64.5^ab^ ±4.7	
** **Severe	68.4^a^ ±2.2		68.4^a^ ±2.3		73.9^a^ ±4.5		70.3^a^ ±4.9	
DASS-S								
** **Normal	60.2^b^ ± 1.0	0.02	61.3^a^ ± 1.2	0.08	67.0^a^ ± 3.9	0.07	64.4^a^ ± 4.3	0.2
** **Mild	60.7^ab^ ± 2.2		60.7^a^ ± 2.3		66.3^a^ ± 4.5		63.2^a^ ± 4.8	
** **Moderate	67.7^a^ ± 2.1		67.4^a^ ± 2.3		73.2^a^ ± 4.5		69.4^a^ ± 4.9	
** **Severe	62.8^ab^ ± 2.7		62.8^a^ ± 2.8		68.4^a^ ± 4.8		65.4^a^ ± 5.1	

Model 1: age and sex; Model 2: age, sex, education, income, and smoking; Model 3: age, sex, education, income, smoking, and BMI

LSM, least squares mean; SEM, standard error of the mean; CI, confidence interval.

Values in the same row (within an analysis) denoted with the same letter are not significantly different from each other (Tukey’s HSD *P* < 0.05).

**Table 7.** Recalled salt intensity does not vary by DASS-21 scores indicative of depression, anxiety, and stress in the total sample (unadjusted and adjusted models).

DASS-21	Recalled salt intensity							
	Unadjusted		Model 1		Model 2		Model 3	
	LSM ±SEM	*P* trend	LSM ±SEM	*P* trend	LSM ±SEM	*P* trend	LSM ±SEM	*P* trend
DASS-D								
** **Normal	53.9^a^ ±1.1	0.6	53.0^a^ ±1.3	0.5	45.6^a^ ±4.1	0.5	43.1^a^ ±4.5	0.4
** **Mild	56.6^a^ ±2.3		56.5^a^ ±2.4		49.2^a^ ±4.7		47.2^a^ ±5.1	
** **Moderate	52.8^a^ ±2.5		53.1^a^ ±2.6		45.4^a^ ±4.8		44.1^a^ ±5.2	
** **Severe	55.1^a^ ±2.4		55.3^a^ ±2.4		47.5^a^ ±4.6		45.5^a^ ±5.1	
DASS-A								
** **Normal	54.5^a^ ±1.0	0.2	53.5^a^ ±1.2	0.3	46.0^a^ ±4.1	0.1	43.5^a^ ±4.5	0.2
** **Mild/Moderate	56.0^a^ ±1.8		56.3^a^ ±2.0		48.6^a^ ±4.4		46.6^a^ ±4.9	
** **Severe	50.6^a^ ±2.3		51.8^a^ ±2.5		42.9^a^ ±4.6		41.3^a^ ±5.1	
DASS-S								
** **Normal	54.9^a^ ±1.1	0.8	53.9^a^ ±1.2	1.0	46.2^a^ ±4.1	1.0	43.7^a^ ±4.5	1.0
** **Mild	52.8^a^ ±2.4		53.0^a^ ±2.5		44.7^a^ ±4.7		42.6^a^ ±5.0	
** **Moderate	52.9^a^ ±2.3		53.7^a^ ±2.4		45.6^a^ ±4.7		43.7^a^ ±5.2	
** **Severe	54.7^a^ ±2.8		54.3^a^ ±3.0		45.6^a^ ±4.9		43.4^a^ ±5.4	

Model 1: age and sex; Model 2: age, sex, education, income, and smoking; Model 3: age, sex, education, income, smoking, and BMI.

LSM, least squares mean; SEM, standard error of the mean; CI, confidence interval.

Values in the same row (within an analysis) denoted with the same letter are not significantly different from each other (Tukey’s HSD *P* < 0.05).

#### 3.5.1 DASS-D and recalled salt liking.

Higher salt liking was associated with DASS-21 scores indicative of depression in the unadjusted and adjusted models (*P* trend range = 0.01–0.04; Table 6). Salt liking was 13.8% higher in those with scores indicative of severe depression than in those with normal scores in the unadjusted model (60.0 vs. 68.3, *P* = 0.005, 95% CI 1.8–14.7; Table 6). Statistical significance was maintained for this relationship after adjusting for age and sex (60.8 vs. 68.3, *P* = 0.02, 95% CI 1.0–13.9), age, sex, income, education, and smoking (66.0 vs. 74.0, *P* = 0.01, 95% CI 1.3–14.7), and age, sex, income, education, smoking, and BMI (63.2 vs. 70.7, *P* = 0.02, 95% CI 0.8–14.1) Table 6).

#### 3.5.2 DASS-A and recalled salt liking.

Higher salt liking was associated with DASS-21 scores indicative of anxiety in the unadjusted and adjusted models (*P* trend range = 0.002–0.03; Table 6). Salt liking was 14% higher in those with scores indicative of severe anxiety than in those with normal scores (60.0 vs. 68.4, *P* = 0.001, 95% CI 2.8–14.0; Table 6). Statistical significance was maintained for this relationship after adjusting for age and sex (61.1 vs. 68.4; *P* = 0.01, 95% CI 1.4–13.1), age, sex, income, education, and smoking (66.4 vs. 73.9; *P* = 0.01, 95% CI 1.5–13.6), and age, sex, income, education, smoking, and BMI (63.4 vs. 70.3; *P* = 0.02, 95% CI 0.8–13.0) Table 6).

#### 3.5.3 DASS-S and recalled salt liking.

Higher salt liking was associated with DASS-21 scores indicative of stress in the unadjusted model (*P* trend = 0.02; Table 6). Salt liking was 12.5% higher in those with scores indicative of moderate stress than in those with normal scores (60.2 vs. 67.7, *P* = 0.009, 95% CI 1.4–13.5; Table 6). Statistical significance was not maintained in the adjusted models.

### 3.6 Relationships between DASS-21 and recalled salt taste liking and intensity, by sex

Females with DASS-21 scores indicative of anxiety and stress had higher mean salt liking scores (Supplementary Table S1). In the crude analyses only, males with indicative depression and anxiety had higher mean salt liking scores (Supplementary Table S1). In females and males, there were no associations between salt intensity and any of the mood states (Supplementary Table S2).

#### 3.6.1 DASS-D and recalled salt liking.

The model *P* trends (unadjusted and adjusted) for the relationships between DASS-21 scores indicative of depression and salt liking were not statistically significant for either females or males (Supplementary Table S1). However, in males salt liking was 19.4% higher in those with DASS-21 scores indicative of severe depression than in those with normal scores in the unadjusted model only (59.7 vs. 71.3, *P* = 0.04, 95% CI 0.2–23.0; Supplementary Table S1).

#### 3.6.2 DASS-A and recalled salt liking.

In females, higher salt liking was associated with DASS-21 scores indicative of anxiety in the unadjusted and adjusted models (*P* trend range = 0.002–0.0005; Supplementary Table S1) and in males, the association was significant in the unadjusted model only (*P* trend = 0.04; Supplementary Table S1).

In females, salt liking was 15.9% higher in those with DASS-21 scores indicative of severe anxiety than in those with mild/moderate scores in the unadjusted model (60.2 vs. 69.8, *P* = 0.004, 95% CI 2.5–16.8; Supplementary Table S1). This association was statistically significant in the models adjusting for age (59.4 vs. 68.9, *P* = 0.005, 95% CI 2.4–16.7; Supplementary Table S1), age, income, education, and smoking (65.4 vs. 74.8, *P* = 0.007, 95% CI 2.2–16.8; Supplementary Table S1), and age, income, education, smoking, and BMI (60.8 vs. 70.0, *P* = 0.003, 95% CI 2.6–15.8; Supplementary Table S1). In females, salt liking was 17.1% higher in those with DASS-21 scores indicative of severe anxiety than in those with normal scores in the unadjusted model (59.6 vs. 69.8, *P* = 0.003, 95% CI 4.1–16.4; Supplementary Table S1). This association was statistically significant in the models adjusting for age (59.8 vs. 68.9, *P* = 0.003, 95% CI 2.6–15.6; Supplementary Table S1), age, income, education, and smoking (65.1 vs. 74.8, *P* = 0.002, 95% CI 3.0–16.5; Supplementary Table S1), and age, education, income, smoking, and BMI (60.8 vs. 70.0, *P* = 0.003, 95% CI 2.6–15.8; Supplementary Table S1).

In males, salt liking was 19.9% higher in those with DASS-21 scores indicative of mild/moderate anxiety than in those with normal scores in the unadjusted model only (61.3 vs. 73.5, *P* = 0.03, 95% CI 0.9–23.4; Supplementary Table S1).

#### 3.6.3 DASS-S and recalled salt liking.

In females, higher salt liking was associated with DASS-21 scores indicative of stress in the unadjusted, age adjusted, and age, income, education, and smoking-adjusted models (*P* trend range = 0.01–0.04; Supplementary Table S1) but not in the fully adjusted model inclusive of BMI. In males, there were no statistically significant relationships.

In females, salt liking was 14.6% higher in those with DASS-21 scores indicative of moderate stress than in those with normal scores in the unadjusted model (59.5 vs. 68.2, *P* = 0.007, 95% CI 1.8–15.7; Supplementary Table S1). This association was statistically significant in the models adjusting for age (59.8 vs. 67.3, *P* = 0.04, 95% CI 0.2–14.6; Supplementary Table S1), and age, income, education, and smoking (65.9 vs. 74.0, *P* = 0.03, 95% CI 0.7–15.4; Supplementary Table S1), but not in the fully adjusted model inclusive of BMI.

## 4. Discussion

This exploratory study aimed to assess relationships between recalled salt taste liking and intensity, and indices of depression, anxiety, and stress. The survey findings demonstrate higher recalled liking of salt index foods when DASS-21 scores indicate severe levels of depression and anxiety, and moderate levels of stress. Females had higher recalled salt liking when DASS-21 scores indicated severe and mild/moderate anxiety, and moderate stress. Males had higher recalled salt liking when DASS-21 scores indicated severe depression and mild/moderate anxiety in the crude analysis only. There were no relationships between recalled intensity ratings of salt index foods and DASS-21 scores for the total sample or by sex. While these novel findings do not demonstrate causation, they indicate that further research is important to determine if salt taste may be related via signaling, genetic or other biological mechanisms, with potential future roles in diagnosis, prevention, or treatment.

Recalled salt liking was 11.8–13.8% higher in participants with DASS-21 scores indicative of severe levels of depression in comparison with those recording normal scores in the crude analysis and adjusted models. A prior study in people with diagnosed major depressive disorder (*n* = 30) similarly found higher salt liking (pleasantness) correlated with longer periods of depression (Büttner et al. 2015). Conversely, no correlation was found between salt liking (pleasantness) and symptoms of depression in a healthy sample (*n* = 33) (Scinska et al. 2004). Factors likely contributing to the different outcomes between the current study and Scinska et al. (2004) include the use of a recall method for taste measures versus a taste test of salty solutions, the use of different depression assessment tools (DASS-21 vs. Beck Depression Inventory [BDI]), and the substantially larger sample size (*n* = 424) of the current study. The DASS-21 is reported to better discriminate between depression and other affective states than the BDI (Lovibond and Lovibond 1995a), and to have predictive power in the diagnosis of depression (Gloster et al. 2008; Bener et al. 2016). The association of recalled salt liking to severe levels of depression (DASS-21) and not moderate or mild levels in this study, suggests those with scores indicative of depression may be clinically depressed. Therefore, the predictive power of the DASS-21 for depression further aligns the results of this study with the positive association between salt liking and depression found in the study executed in a clinical setting using salt solutions (Büttner et al. 2015). Further studies in both clinical and non-clinical populations would be beneficial in elucidating findings and providing direction for settings most suited to their application.

Recalled salt liking was 10.9–14% higher in those with DASS-21 scores indicating severe levels of anxiety in comparison with those recording normal scores in the crude analysis and adjusted models. Previously, a negative correlation has been found between salt liking (pleasantness) and anxiety in people with major depressive disorder (Büttner et al. 2015). In the study by Büttner et al. (2015), anxiety was measured with 1 question (Item 10) from the Hamilton Depression Rating Scale, an instrument primarily designed to assess symptoms of depression, not anxiety (Carmody et al. 2006). Therefore, the use of DASS-21 in the present study has likely measured scores of anxiety more accurately and reliably. Other research has shown that salt taste thresholds (the concentrations at which saltiness is detected) are significantly higher in anxiety than in healthy samples (Heath et al. 2006; Ileri-Gurel et al. 2013) and that salt liking is positively correlated to salt taste thresholds (Hayes et al. 2010; Chamoun et al. 2019; Puputti et al. 2019; Pilic et al. 2020). These previous studies indicate salt liking may increase in anxiety and the present study supports this theory. However, the prior studies assessed salt taste using salt solutions at varying threshold concentrations including suprathreshold levels therefore the comparison to the outcomes of the current study using recall and salt index foods is hypothetical and requires further exploration.

Recalled salt liking was 12.5% higher in those with DASS-21 scores indicating moderate stress levels however, when adjusting for the confounding variables there was no statistical significance. Complexity in the relationship between salt liking and stress has been demonstrated in a small (*n* = 38) sample of younger (20.3 years) people with no history of mental health disorders, where the physiological measure of stress, cortisol, was not associated to salt liking but there was a negative correlation between self-reported distress after induced stress (Al’Absi et al. 2012). The reasons for the varied outcomes are unclear. However, as salt thresholds and liking are negatively correlated (Hayes et al. 2010; Pilic et al. 2020), the higher salt liking may be a result of a lowering of salt taste thresholds previously demonstrated in stress (Ileri-Gurel et al. 2013). As the most under-researched relationship, salt liking and stress require more robust exploratory and mechanistic investigations using both salty solutions and tasting of salty foods.

The results of the analyses for recalled salt liking by sex has highlighted some differences to those in the total sample. While an association was found between higher recalled salt liking and depression in the total sample the relationship was only found in males (crude analysis), and not in females. Salt liking was higher in those with severe versus normal anxiety in the total sample, in those with severe versus mild/moderate and normal anxiety in females, and in those with mild/moderate anxiety versus normal in males (crude analysis). The finding of higher salt liking in moderate stress was found in the crude analysis in the total sample and in females in all models with the inclusion of BMI as the exception. These varying outcomes may be due to the predominantly female composition of the study sample however this does not explain the differences completely. Previous studies on salt taste qualities and mood (Scinska et al. 2004; Al’Absi et al. 2012; Büttner et al. 2015) did not analyze data by sex so comparative discussion is not possible. However, both salt taste qualities (Hayes et al. 2010; Calvo and Egan 2015; Martin and Sollars 2017 ) and mood (Eid et al. 2019; Rubinow and Schmidt 2019; Seney and Sebille 2014) are known to vary between the sexes therefore there may be sex-hormone mediated roles influencing the relationships that need to be explored.

Recalled salt taste intensity was not associated with depression, anxiety, or stress in the total sample or by sex. Similar to liking, prior research on these relationships has measured taste intensity through taste tests using salty solutions often at supraphysiological concentrations (Scinska et al. 2004; Al’Absi et al. 2012; Itoh et al. 2022). This may account for the varying outcomes in relation to the present study that used recall taste measures. For example, reduced salt intensity (solutions) has been correlated with the rise in cortisol occurring during acute stress (Al’Absi et al. 2012) and to increasing levels of self-reported stress (Itoh et al. 2022). In contrast, the lack of association between recalled salt taste intensity and depression in the present study was also found in a healthy sample of older adults (63 years) that rated the intensity of salty solutions (Scinska et al. 2004). The robustness of the present result is increased by the better discriminatory and diagnostic power for depression of the DASS-21 versus the BDI (Lovibond and Lovibond 1995a; Gloster et al. 2008; Bener et al. 2016) used by Scinska et al. (2004). Additional reasons for these varied outcomes may be differences in the physiology of acute and chronic stress, and use of different mood assessment measures. Finally, differentiating between intensities of varying salt solutions is more difficult than other tastes (Puputti et al. 2018) and recall of taste intensity is likely more challenging than recalling the hedonic experience of taste liking.

Several physiological factors may explain the findings from this study and highlight possible avenues for application and future research. High salt concentrations modulate salt taste receptor release of 5-HT and NE (Roper and Chaudhari 2017), neurotransmitters altered in depression (Dean and Keshavan 2017), and anxiety (Kaur and Singh 2017). If salt taste alterations are indicative of neurotransmitter imbalance, taste tests may be utilized in progress monitoring or diagnosis. Genetic polymorphisms found in the salt taste receptor TRPV1 are associated with altered salt taste sensitivity and preference (Dias et al. 2013; Chamoun et al. 2018; Pilic and Mavrommatis 2018) and to reduced anxiolytic behaviors and antidepressant effects in mice (Marsch et al. 2007). Whether variations in genes coding for salt taste receptors are associated with depression, anxiety, and stress is yet to be elucidated but may be useful in improving their detection, prevention, or treatment. Low serum sodium levels have been linked to depression (Grippo et al. 2006; Morris et al. 2006, 2008, 2010; Özdemir et al. 2014; Sawant et al. 2019), and correction of hyponatraemia can improve symptoms (Sawant et al. 2019; Bojdani et al. 2020). Therefore, salt liking potentially increases in depression in response to low serum sodium or the reduced physiological impacts of serum sodium. For example, increased salt liking under sodium depletion (Tindell et al. 2006) is reflected by increased neuronal activity in the VP the center of hedonic liking in the brain (Smith et al. 2009; Root et al. 2015). Increased firing of VP neurons is also found in depression and silencing of VP neurons can ameliorate depressive-like behaviors (Knowland et al. 2017). Increased salt intake driven by liking of salt (Hayes et al. 2010; Pilic et al. 2020) may lead to TN nerve stimulation (Simon et al. 2008; Bigiani, 2020, 2021) which ameliorates depression (Schrader et al. 2011; Cook et al. 2013; Paulino Trevizol and Cordeiro 2016; Generoso et al. 2019) and anxiety (Trevizol et al. 2015; Freire et al. 2020). Liking of salty foods can be reduced through exposure to lower sodium diets (Riis et al. 2021). Therefore, it is possible that a dietary intervention reducing liking of salty foods may increase TN nerve stimulation improving anxiety and depression or may reduce VP neuronal firing improving depression.

This study is the first to find associations between recalled liking of salt-index foods and DASS-21. A conservative approach to generalizing these findings is recommended as the sample was predominantly female and highly educated with incomes above average. However, none of the demographic variables influenced the statistically significant relationships in depression and anxiety. The larger numbers of participants experiencing some level of depression, anxiety, and stress (~36%) added power to the data and is somewhat reflective of early evidence of the increased prevalence of mental health issues reported during the COVID-19 pandemic when this study was undertaken (Morgan 2020; Aknin et al. 2022). Surveys are susceptible to sampling, response bias, and non-response errors but also have advantages such as removing interviewer bias and providing anonymity to participants (Fricker and Schonlau 2002; Van Selm and Jankowski 2006). In this case, the survey format allowed for the capture of a large sample size and broader experiences of recalled salt taste measures and mood not previously recorded. Questionnaires are an appropriate method for population studies such as this (Leshem 1998; Keskitalo et al. 2007; Cornelis et al. 2017; Wanich et al. 2018), and have been moderately correlated with outcomes from laboratory studies that measure the more sensory experience of food (Deglaire et al. 2012). The use of salt-index foods rather than supraphysiological salt solutions to obtain recall measures of salt liking and intensity allows for the results to be considered in real-world settings. The use of multiple salty foods to arrive at means for recalled salt liking and intensity reduced the influence that interindividual differences between participants’ taste experiences may exert on outcomes (Cornelis et al. 2017). However, the recall method used in this study to assess the salt taste qualities is highly subjective and the next steps in research should include the tasting of salt index foods in a clinical setting.

The aim of this cross-sectional study was to explore the relationships between aspects of salt taste and mood. The novel findings demonstrate that recalled liking of salt index foods increases when DASS-21 scores indicate severe depression and anxiety, and moderate stress. Further examination of the relationships between salt liking and mental health and the mechanisms that may be involved in the associations would be beneficial to proving/disproving these findings and moving toward their appropriate application in clinical and non-clinical settings. Future research should include tasting of salty foods in clinical settings, data on salt intake, serum sodium levels, genetics, and ethnicity, and have a more even sex distribution. Interventions that measure neurotransmitter levels and nervous system responses common to both salt taste and depression, anxiety, and stress would be valuable to understand the mechanisms involved in the correlations found and better direct any potential new assessment methods or treatments.

## Supplementary Material

bjad038_suppl_Supplementary_Tables_S1-S2

## Data Availability

The raw data supporting the conclusions of this article will be made available by the authors, without undue reservation.
